# Oleanolic acid and its derivatives in breast cancer therapy: mechanistic insights, structural modifications, and novel delivery strategies

**DOI:** 10.3389/fphar.2026.1755987

**Published:** 2026-03-02

**Authors:** Xiaofei Hu, Yuting Qin

**Affiliations:** Shandong University of Traditional Chinese Medicine, Jinan, Shandong, China

**Keywords:** antitumor mechanisms, breast cancer, novel delivery strategies., oleanolic acid, oleanolic acid derivatives, structural modifications

## Abstract

Oleanolic acid is a natural pentacyclic triterpenoid widely found in dietary plants and has attracted translational interest in breast cancer research. This review addresses how oleanolic acid and its derivatives exert anticancer effects in breast cancer models, and how structural modification and delivery strategies may mitigate key barriers that currently limit clinical translation. Drawing primarily on *in vitro* studies and preclinical animal evidence, we summarize major mechanistic themes, including induction of apoptosis and cell-cycle arrest, and regulation of autophagy, ferroptosis, oxidative stress, cancer metabolism, and tumor microenvironment–associated processes. We further highlight representative medicinal-chemistry advances showing that selected derivatives, such as SZC014 and HIMOXOL, can exhibit improved physicochemical properties and enhanced anticancer activity in specific breast cancer models. In parallel, we review formulation and delivery approaches aimed at improving exposure and tumor delivery, including nanoparticle-based systems and emerging co-delivery or self-assembly strategies developed to address poor solubility and limited bioavailability. Importantly, we critically discuss why pharmacokinetic limitations remain a central obstacle, including poor aqueous solubility, variable absorption, extensive first-pass metabolism, uncertain tumor exposure, and limited PK/PD linkage. We also note that derivatization and nanocarriers may introduce new uncertainties related to metabolic fate, drug–drug interactions, off-target accumulation, manufacturability, and long-term safety. Across the field, conclusions are additionally constrained by model heterogeneity, incomplete subtype coverage, limited normal-cell controls, and insufficient mechanistic causality testing. Overall, oleanolic acid and its derivatives should be viewed as preclinical leads with potential relevance to breast cancer, and future progress will require standardized multi-model validation, rigorous PK and biodistribution profiling with PK/PD integration, systematic toxicology, and rational combination strategies before clinical utility can be concluded.

## 1 Introduction

Breast cancer is one of the most common malignant tumors among women worldwide (Kaya et al., 2025; Giannakeas et al., 2024). In 2022, the World Health Organization (WHO) reported approximately 2.3 million new cases and 670,000 deaths (Xiong et al., 2025). It is highly heterogeneous and is commonly stratified by estrogen receptor (ER), progesterone receptor (PR), human epidermal growth factor receptor 2 (HER2), and Ki-67 status into luminal A, luminal B, HER2-positive, and triple-negative breast cancer (TNBC) subtypes (Xiong et al., 2025; Jafarinejad-Farsangi et al., 2022; Prat et al., 2015; Asleh et al., 2022). Despite advances in surgery, radiotherapy, chemotherapy, and targeted or immunotherapeutic approaches (Maughan et al., 2010; Sarhangi et al., 2022; Sachdev et al., 2019), treatment resistance and adverse effects remain major challenges, highlighting the need for safer and more effective therapies (Arnedos et al., 2015; Braga, 2016).

In recent years, the potential of natural products in cancer therapy has gained increasing attention (Zhang et al., 2025; Li et al., 2025; Chen T. et al., 2024; Tewari et al., 2022). Oleanolic acid (OA) is a naturally occurring pentacyclic triterpenoid widely distributed in plants and has demonstrated antitumor activity through multi-target and multi-pathway mechanisms (Pollier and Goossens, 2012; Žiberna et al., 2017; Zeng et al., 2025). However, the clinical translation of OA is limited by poor drug-like properties, particularly strong hydrophobicity and extremely low water solubility, which contribute to low bioavailability and suboptimal tumor exposure (Castellano et al., 2022; Sultana and Ata, 2008). To overcome these barriers and enhance pharmacological performance, extensive efforts have focused on the design of OA derivatives through semi-synthesis and structural modification (Meng et al., 2019; Staicu et al., 2024; Triaa et al., 2024). Representative strategies include introducing electrophilic “warheads” to strengthen target engagement, forming oxime or related functional groups to modulate polarity and reactivity, and constructing ring-fused or heterocycle-containing analogs to improve potency and selectivity, yielding derivatives such as SZC014 and HIMOXOL that show enhanced anticancer activity in specific breast cancer models. In parallel, OA derivatives are increasingly developed alongside advanced formulation approaches to further improve delivery and therapeutic windows. More broadly, pentacyclic triterpenoids have been reported to influence multiple stages of tumor initiation and progression by inhibiting cancer cell proliferation, inducing apoptosis, suppressing angiogenesis, and limiting invasion and metastasis, supporting their continued investigation as anticancer scaffolds (Staicu et al., 2024; Wiemann et al., 2016; Baer-Dubowska et al., 2021).

This review primarily focuses on recent advances in the study of OA and its derivatives in breast cancer, providing a systematic summary of their mechanisms of action, structural modifications, and innovative drug delivery strategies, while also discussing current challenges and future prospects for clinical application.

## 2 Literature search strategy

This review employed a structured literature search to systematically identify studies on OA and its derivatives in breast cancer, with a focus on antitumor mechanisms, structural modifications, and drug delivery strategies. Searches were conducted in PubMed, Web of Science Core Collection, ScienceDirect, and Scopus from database inception through November 2025. The search terms covered OA- and breast cancer–related concepts, including “oleanolic acid,” “derivative,” “triterpenoid,” “breast cancer,” “MCF-7,” “MDA-MB-231,” “triple-negative,” and “HER2.” Reference lists of included articles and key reviews were also hand-searched to capture additional relevant studies not retrieved through database queries.

Eligible records were limited to peer-reviewed, English-language publications that met at least one of the following criteria: investigation of OA or OA derivatives in breast cancer–relevant *in vitro* or *in vivo* models; provision of mechanistic evidence; or evaluation of formulations and delivery approaches intended to improve systemic exposure or tumor targeting. Studies unrelated to breast cancer, lacking OA-based interventions, or focused solely on botanical extraction and analytical characterization without therapeutic relevance were excluded. All records were de-duplicated, screened by title and abstract, and subsequently assessed in full text. Given the heterogeneity of experimental models and outcome measures, findings were synthesized narratively rather than by meta-analysis, with particular attention to result consistency, subtype context, dose comparability, and the adequacy of pharmacokinetic and safety evidence.

## 3 Antitumor mechanisms of oleanolic acid in breast cancer

### 3.1 Induction of apoptosis

**FIGURE 1:**
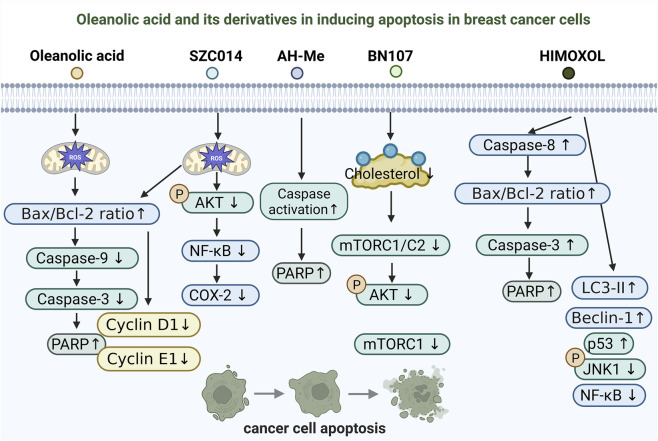
Proposed mechanisms of apoptosis induction by oleanolic acid and its derivatives in breast cancer cells. Schematic overview of apoptosis-related pathways reported for oleanolic acid (OA) and representative derivatives (SZC014, AH-Me, BN107, and HIMOXOL) in breast cancer models. OA and SZC014 are associated with ROS-linked mitochondrial apoptosis, reflected by an increased Bax/Bcl-2 ratio, caspase cascade engagement, and increased PARP cleavag, with OA also reducing Cyclin D1/E1. SZC014 further inhibits the Akt-NF-κB/COX-2 axis. AH-Me induces caspase-dependent apoptosis, BN107 is linked to cholesterol depletion with mTORC1/C2 and Akt inhibition, and HIMOXOL activates caspase-8–associated apoptosis with crosstalk to LC3-II/Beclin-1 and modulation of NF-κB, p53, and JNK signaling.

Inducing apoptosis in tumor cells is a central mechanism underlying the action of many anticancer agents (Moyer et al., 2025; Schmitt, 2003). One of the key anticancer activities of OA and its derivatives in breast cancer therapy lies in their ability to trigger apoptotic cell death (Figure 1). Multiple studies have demonstrated that OA and its structurally modified analogs can activate apoptosis through various signaling pathways, accompanied by characteristic mitochondrial damage and caspase cascade activation (Chu et al., 2017; Fukumura et al., 2009; Chakravarti et al., 2012; Chu et al., 2010; Lisiak et al., 2014).

The synthetic OA derivative SZC014 has been shown to markedly inhibit proliferation and migration of MCF-7 breast cancer cells by upregulating the Bax/Bcl-2 ratio, activating caspase-9/3, and promoting PARP cleavage, thereby inducing mitochondria-dependent apoptosis. This effect is mediated by excessive accumulation of reactive oxygen species (ROS), as pretreatment with N-acetylcysteine (NAC) reverses the apoptotic response, indicating that ROS serve as a critical upstream signal. In addition, SZC014 suppresses Akt phosphorylation and blocks the NF-κB/COX-2 signaling pathway, suggesting a ROS-dependent, multitarget mechanism of apoptosis induction (Chu et al., 2017).

Similarly, the OA-based saponin derivative Achyranthoside H methyl ester (AH-Me) exerts potent cytotoxic effects against MCF-7 and MDA-MB-453 breast cancer cells by activating a caspase-dependent apoptotic pathway characterized by nuclear fragmentation, an increase in sub-G1 phase cells, and PARP cleavage. This cytotoxic effect can be reversed by caspase inhibitors (Fukumura et al., 2009). Ethanol extracts from *Wrightia tomentosa* and their active fraction F-4 also exhibit pro-apoptotic activity in breast cancer cells. Further analysis identified OA and ursolic acid as the principal active constituents responsible for the decline in mitochondrial membrane potential, elevated ROS generation, activation of caspase-8, and an increased Bax/Bcl-2 ratio, collectively triggering apoptosis (Chakravarti et al., 2012).

Another study revealed that OA can also induce apoptosis by modulating cell membrane structure and energy metabolism pathways. BN107, a plant extract rich in OA, selectively depletes raft-associated cholesterol in ER-negative breast cancer cells, leading to disruption of raft-dependent survival signaling, dual inhibition of mTORC1/C2, and inactivation of Akt, ultimately resulting in apoptosis. This effect is more pronounced in ER-negative cells, while supplementation with exogenous cholesterol significantly attenuates the apoptotic response (Chu et al., 2010).

Moreover, some OA derivatives display dual regulation of apoptosis and other forms of programmed cell death. The synthetic derivative HIMOXOL exhibits stronger cytotoxicity than the parent compound in TNBC MDA-MB-231 cells. Mechanistically, HIMOXOL activates the extrinsic caspase-8 pathway, disrupts the Bax/Bcl-2 balance, and induces PARP cleavage, accompanied by autophagosome formation and elevated expression of LC3-II and Beclin-1. Additionally, HIMOXOL modulates MAPK and p53 signaling while suppressing NF-κB activity, indicating that it exerts antitumor effects through a dual mechanism involving both apoptosis and autophagy (Lisiak et al., 2014).

OA and its derivatives induce apoptosis in breast cancer cells through multiple mechanisms, including ROS generation, mitochondrial pathway activation, caspase-dependent cascades, lipid raft cholesterol depletion, and modulation of key signaling pathways (Chu et al., 2017; Fukumura et al., 2009; Chakravarti et al., 2012; Chu et al., 2010). Collectively, these studies help clarify the mechanistic breadth of OA’s anticancer activity and provide a rationale for developing more selective and potent OA-based candidates. However, a key challenge in interpreting the literature is that most evidence is derived from a limited set of breast cancer cell lines and short-term xenograft models that do not fully capture clinical heterogeneity (McDonough et al., 2024; LeSavage et al., 2022). Because molecular subtypes defined by ER, PR, HER2, and proliferative status differ in redox balance, metabolic dependencies, and susceptibility to regulated cell death, reported effects such as ROS-associated apoptosis, AMPK activation, PI3K/Akt/mTOR inhibition, and crosstalk with autophagy or ferroptosis may not generalize across contexts or dosing windows (Das et al., 2024; Carvalho et al., 2025; Bel’skaya and Dyachenko, 2024). Moreover, non-standardized concentrations, exposure durations, and formulations, together with underreporting of negative results, can confound comparisons and bias interpretations toward overly coherent mechanistic narratives (Carpi et al., 2024; Cobey et al., 2024). Future studies should therefore prioritize standardized, head-to-head testing across subtype-representative models with normal breast epithelial controls, and incorporate quantitative exposure–response and mechanistic readouts to improve reproducibility and translational relevance.

### 3.2 Autophagy and ferroptosis

**FIGURE 2:**
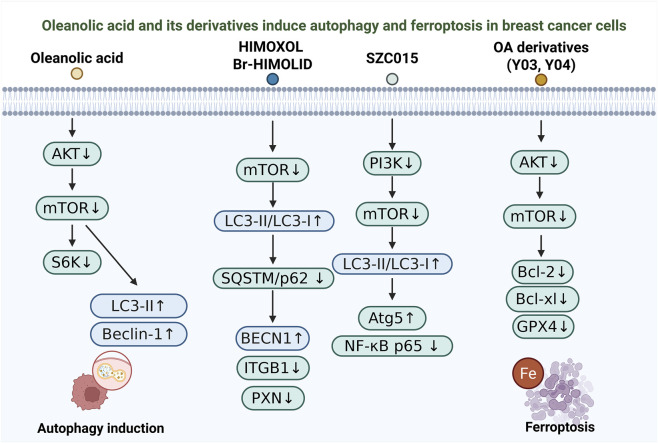
Oleanolic acid and its derivatives induce autophagy and ferroptosis in breast cancer cells. Schematic overview of reported pathways by which oleanolic acid (OA) and representative derivatives regulate autophagy and ferroptosis in breast cancer models. OA suppresses the Akt–mTOR–S6K axis and is associated with increased autophagy markers (LC3-II and Beclin-1). HIMOXOL/Br-HIMOLID inhibit mTOR and are linked to an increased LC3-II/LC3-I ratio, reduced SQSTM1/p62, increased BECN1, and downregulation of ITGB1 and PXN. SZC015 is associated with PI3K–mTOR modulation, increased LC3-II/LC3-I and Atg5, and decreased NF-κB p65. Ferroptosis-oriented OA derivatives suppress Akt–mTOR signaling and downregulate Bcl-2, Bcl-xL, and GPX4, consistent with ferroptotic cell death (Fe).

In addition to inducing apoptosis, OA and its derivatives exert anti-breast cancer effects by triggering autophagy and ferroptosis (Figure 2). During tumor progression, autophagy functions as a “double-edged sword,” capable of promoting cell survival under stress but also inducing cell death under specific conditions (Debnath et al., 2023; Miller and Thorburn, 2021). Meanwhile, ferroptosis—an iron-dependent, non-apoptotic form of regulated cell death—has attracted increasing attention due to its unique ability to selectively target and eliminate drug-resistant cancer cells (Mou et al., 2019; Zhang et al., 2022; Zhou Q. et al., 2024).

OA itself has been shown to induce non-cytotoxic autophagy in multiple cell types by suppressing activation of the Akt/mTOR/S6K signaling pathway, thereby enhancing autophagic flux (Liu et al., 2014a). In KRAS-mutated transformed cells, OA-induced autophagy markedly inhibits cell proliferation, invasion, and anchorage-independent growth, while treatment with autophagy inhibitors partially reverses these effects, suggesting that OA modulates the malignant phenotype of precancerous cells through autophagy regulation.

Semi-synthetic OA derivatives such as HIMOXOL and Br-HIMOLID exhibit potent cytotoxicity and anti-migratory activity in HER2-positive breast cancer cells (Lisiak et al., 2021). Their mechanisms are closely associated with autophagy activation mediated by the mTOR/LC3/SQSTM1/BECN1 signaling axis, accompanied by cell cycle arrest and mild apoptosis, indicating that autophagy induction plays a central role in their antitumor effects. Another derivative, SZC015, displays a more complex mechanism by concurrently inducing both apoptosis and autophagy (Wu et al., 2016). The apoptotic process involves mitochondrial pathway activation and caspase cleavage, whereas autophagy is characterized by LC3-II accumulation and Beclin-1 upregulation. Furthermore, SZC015 inhibits PI3K/Akt/mTOR/NF-κB and MAPK pathways and exhibits topoisomerase inhibitory activity, suggesting a synergistic, multi-target mode of action in promoting breast cancer cell death.

Beyond autophagy, OA-related derivatives have also expanded into the realm of ferroptosis-based antitumor therapy (Sun et al., 2025; Xiaofei et al., 2021). Studies have shown that OA derivatives containing electrophilic warheads, such as Y03 and Y04, significantly suppress GPX4 activity in breast cancer cells, thereby initiating ferroptosis (Wang et al., 2023). These compounds display potent cytotoxicity at submicromolar concentrations, accompanied by ROS accumulation, inhibition of the Akt/mTOR pathway, and downregulation of anti-apoptotic proteins, highlighting ferroptosis as a promising and emerging mechanism of OA-derived anticancer activity.

OA and its derivatives exhibit multifaceted antitumor activities in breast cancer cells, in part by engaging autophagy and ferroptosis in a context-dependent manner (Liu et al., 2014a; Lisiak et al., 2021; Wu et al., 2016; Sun et al., 2025; Xiaofei et al., 2021). Together, these findings expand the mechanistic landscape of OA-based compounds and support their continued exploration as multi-target therapeutic candidates for breast cancer. However, current interpretations are constrained by the frequent reliance on autophagy markers such as LC3-II and Beclin-1, which alone cannot distinguish increased autophagic flux from impaired lysosomal degradation (Klionsky et al., 2021; Liebl et al., 2022). Moreover, because autophagy can be either cytoprotective or cytotoxic depending on cellular context, apparent “autophagy activation” may contribute to divergent outcomes across cell lines and dosing windows (Ahmadi-Dehlaghi et al., 2023; Kwantwi, 2024; Jain et al., 2023). For ferroptosis, GPX4 suppression and ROS elevation are suggestive but insufficient for definitive assignment, particularly for multi-target derivatives that also perturb Akt/mTOR signaling and apoptotic regulators, making mixed death phenotypes likely (Liu et al., 2025; Chen X. et al., 2024). Standardized flux measurements and orthogonal ferroptosis validation are therefore needed to establish causality and improve reproducibility (Thi Nghiem et al., 2025).

### 3.3 Cell cycle arrest

**FIGURE 3:**
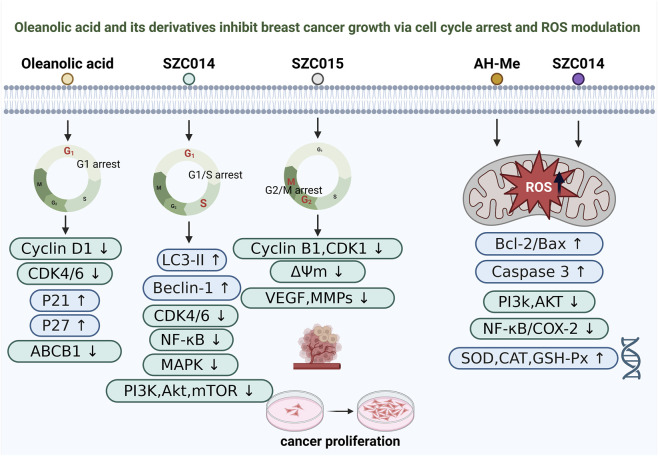
Oleanolic acid and its derivatives inhibit breast cancer growth via cell-cycle arrest and ROS modulation. Schematic overview of reported mechanisms by which oleanolic acid (OA) and representative derivatives suppress breast cancer cell proliferation. OA is associated with G_1_ arrest accompanied by Cyclin D1 and CDK4/6 downregulation, p21 and p27 induction, and reduced ABCB1. SZC014 is linked to G_1_/S arrest and is associated with increased LC3-II and Beclin-1 and inhibition of CDK4/6, NF-κB, MAPK, and PI3K/Akt/mTOR signaling. SZC015 is associated with G_2_/M arrest together with reduced Cyclin B1 and CDK1, loss of mitochondrial membrane potential (ΔΨm), and decreased VEGF and MMPs. In parallel, OA, AH-Me, and SZC014 modulate redox-related pathways, including altered mitochondrial ROS, regulation of Bcl-2/Bax and caspase-3, suppression of PI3K/Akt and NF-κB/COX-2 signaling, and induction of antioxidant enzymes (SOD, CAT, and GSH-Px).

The antitumor effects of OA and its derivatives in breast cancer cells are not only mediated by apoptosis and autophagy but are also closely associated with cell cycle arrest (Figure 3). Inducing cell cycle arrest directly inhibits tumor cell proliferation by preventing mitotic progression, thereby serving as a critical mechanism in tumor growth suppression (Glaviano et al., 2025; Fuentes-Antrás et al., 2024).

Existing studies have demonstrated that OA and its derivatives can target multiple cell cycle checkpoints under distinct molecular contexts, thereby suppressing cancer cell proliferation and survival. Regarding G_1_ phase arrest, the novel derivative SZC014 significantly inhibits the proliferation of MCF-7 cells and induces G_1_ phase arrest, accompanied by the activation of apoptosis-related molecules (Chu et al., 2017). Similarly, the parent compound OA induces G_1_ arrest in multidrug-resistant human cancer cells (MCF-7/doxorubicin (Dox) and HL-60/HAR) and reduces ABCB1 protein levels, suggesting its potential in reversing chemoresistance (Wang et al., 2021).

In terms of G_2_/M phase arrest, a polyamine-anchored nanocrystalline capsule (Put-D + O@NCs) was developed to co-deliver Dox and OA. This formulation effectively induces G_2_/M phase arrest in MDA-MB-231 cells, accompanied by mitochondrial membrane depolarization and apoptosis (Shukla et al., 2021). Moreover, both *in vitro* and *in vivo* experiments have shown that this nanosystem exhibits potent antiangiogenic and antimetastatic effects, offering a novel delivery approach for OA-based combination therapy.

As for S phase arrest, the A-ring fused pyrimidine derivative (compound 10) exhibits comparable activity to Dox in MCF-7 cells, significantly inducing S phase arrest and apoptosis (Mallavadhani et al., 2014). Another derivative, SZC015, also triggers S phase arrest in MCF-7 cells, along with the activation of apoptosis and autophagy and inhibition of the PI3K/Akt/mTOR/NF-κB and MAPK signaling pathways (Wu et al., 2016).

In summary, OA and its derivatives can suppress breast cancer cell proliferation by arresting the cell cycle at different checkpoints, including G_1_, G_2_/M, and S phases (Wang et al., 2021; Shukla et al., 2021; Mallavadhani et al., 2014). These observations broaden the mechanistic framework for OA-based antitumor activity and provide a rationale for the rational design of OA-derived agents and combination regimens (Similie et al., 2024; D'Mello et al., 2025). However, cell-cycle arrest may also arise secondarily from generalized cytotoxic stress, metabolic disruption, or DNA damage, so checkpoint accumulation alone does not establish direct engagement of specific cyclin–CDK programs (Helleday and Rudd, 2022; Stallaert et al., 2022; Yam et al., 2022). In co-delivery systems, the relative contribution of OA versus the partner drug is often not resolved with matched single-agent and dose-equivalent controls (Gao et al., 2022; Mao and Guo, 2023). Incorporating durable endpoints such as clonogenic survival and tracking resistance evolution would therefore strengthen mechanistic attribution and translational relevance (Aalam et al., 2024).

### 3.4 Modulation of reactive oxygen species

ROS play a dual role in the initiation, progression, and drug sensitivity of breast cancer, functioning both as signaling molecules that promote tumor development and, when excessively accumulated, as inducers of apoptosis (Nakamura and Takada, 2021; O'Reilly et al., 2024; Moloney and Cotter, 2018). Multiple studies have revealed that OA and its derivatives exert antitumor effects against breast cancer by modulating intracellular ROS levels (Chu et al., 2017; Fukumura et al., 2009; Sánchez-Quesada et al., 2015) (Figure 3).

Firstly, the novel OA derivative SZC014 exhibits potent antiproliferative and pro-apoptotic activity in MCF-7 cells, which is largely dependent on the excessive generation of ROS (Chu et al., 2017). ROS act as upstream signals to activate the mitochondrial apoptotic pathway, characterized by an increased Bax/Bcl-2 ratio, activation of Caspase-9 and Caspase-3, and PARP cleavage (Cheung and Vousden, 2022; Liu et al., 2023; Wang et al., 2024). These events are accompanied by inhibition of key oncogenic signaling pathways such as PI3K/Akt and NF-κB/COX-2, resulting in a multitargeted anticancer effect (Anastasiou et al., 2024; Mishra et al., 2021). Secondly, AH-Me, an OA derivative isolated from Achyranthes fauriei, also mediates its antitumor effects through ROS-related mechanisms. In both MCF-7 and MDA-MB-453 cells, AH-Me induces classical apoptotic features, such as chromatin condensation and nuclear fragmentation, through a caspase-dependent pathway, resulting in pronounced antiproliferative and pro-apoptotic effects (Fukumura et al., 2009). Moreover, the natural triterpenoid OA itself demonstrates a context-dependent dual regulatory effect on oxidative stress (Su et al., 2018; Cheng et al., 2023). In non-cancerous mammary epithelial cells MCF10A, OA acts as an antioxidant, reducing ROS levels and protecting DNA from oxidative damage. Conversely, in breast cancer cells such as MDA-MB-231, OA promotes ROS accumulation, leading to DNA damage and inhibition of cell proliferation (Sánchez-Quesada et al., 2015). This selective redox modulation suggests OA possesses a unique “protect normal cells, kill cancer cells” therapeutic potential.

OA and its structurally modified derivatives exert multifaceted effects in breast cancer by modulating ROS generation and clearance (Chu et al., 2017; Wang et al., 2023; Sánchez-Quesada et al., 2015). This redox regulation may contribute to mitochondrial apoptosis and has also been linked to context-dependent responses, with antioxidative effects reported in normal cells and pro-oxidative stress observed in malignant cells (Chu et al., 2017; Malla et al., 2021). Collectively, these findings suggest that OA could be explored as a candidate for breast cancer chemoprevention and redox-oriented therapy. However, ROS readouts are highly sensitive to baseline redox status, culture conditions, probe specificity, and sampling time points, which complicates cross-study comparisons and may underlie inconsistent results (Kalyanaraman et al., 2020; Wardman, , 2023; Murphy et al., 2022). Moreover, the apparent selectivity between malignant and non-malignant cells has not been robustly validated across broader normal breast epithelial models and diverse breast cancer subtypes (Qu et al., 2015; Gambardella et al., 2022; Maycotte et al., 2024). Increased ROS may also arise secondarily from mitochondrial dysfunction or metabolic stress, and antioxidant rescue experiments may not establish causality when parallel death pathways are engaged (Bel’skaya and Dyachenko, 2024). Therefore, standardized ROS quantification and subtype-stratified validation are needed before OA can be positioned as a reliable redox-targeting strategy (Oshi et al., 2022).

### 3.5 Regulation of cancer metabolism

Metabolic reprogramming is a hallmark of tumor cells, enabling rapid proliferation and survival, and is particularly characterized by enhanced glycolysis and aberrantly active lipid and protein biosynthesis (Halma et al., 2023; Tufail et al., 2024). Recent studies have indicated that OA can modulate cancer cell energy metabolism through multiple signaling pathways, thereby suppressing tumor growth (Figure 3).

On one hand, OA has been reported to promote metabolic reprogramming in breast cancer cells, and studies in MCF-7 suggest that this effect is mediated by activation of the AMPK pathway (Liu et al., 2014b). OA was associated with suppression of lipid synthesis, indicated by downregulation of ACC, FASN, and HMGR, and inhibition of mTOR-dependent protein synthesis via the mTOR p70S6K 4E-BP1 cascade (Ganapathy-Kanniappan and Geschwind, 2013). In parallel, OA reduced glucose uptake and lactate production while increasing oxygen consumption, consistent with a metabolic shift from glycolysis toward oxidative phosphorylation. Mechanistic interventions using AMPK inhibitors and mTOR activators further supported AMPK activation as a key upstream driver of these metabolic changes and the resulting antitumor effects in MCF-7 cells (Ganapathy-Kanniappan and Geschwind, 2013).

On the other hand, OA can directly interfere with glycolysis by inhibiting the mTOR/c-Myc/PKM2 axis (Liu et al., 2014c). OA downregulates c-Myc and its targets hnRNPA1/2, promoting alternative splicing of the PKM gene from the PKM2 isoform to PKM1. This switch reduces aerobic glycolysis while enhancing oxidative phosphorylation, leading to decreased glucose consumption and lactate production, increased oxygen utilization, and ultimately inhibition of cancer cell proliferation (Chelakkot et al., 2023). Overexpression of PKM2 partially reverses OA’s anti-metabolic and antitumor effects, highlighting the PKM2-dependence of its metabolic reprogramming activity.

OA appears to modulate tumor metabolic networks through a coordinated, multi-target program that involves activation of the AMPK pathway and inhibition of the mTOR/c-Myc/PKM2 signaling axis (Liu et al., 2014b; Ganapathy-Kanniappan and Geschwind, 2013; Liu et al., 2014c). Together, these actions can constrain biosynthetic energy supply from lipid and protein synthesis as well as glycolysis, while promoting a shift toward oxidative metabolism, supporting OA as a natural compound with metabolism-targeting potential in cancer cells. However, many metabolic inferences are based on enzyme expression changes and surrogate functional measures such as glucose uptake, lactate production, and oxygen consumption, all of which are sensitive to nutrient availability, cell density, and mitochondrial fitness (Divakaruni and Jastroch, 2022). In addition, metabolic dependencies differ substantially across breast cancer subtypes, so results from a limited set of models may not generalize to clinically diverse tumors (Fukano et al., 2021). Finally, pathway crosstalk among AMPK activation, ROS stress, and cell-cycle regulation can yield similar downstream phenotypes, complicating causal attribution to a single metabolic axis (Kazyken et al., 2024). Importantly, some *in vitro* effects may require exposures that are difficult to achieve in tumors given OA’s pharmacokinetic constraints, highlighting the need for integrated PK/PD analyses and flux-based validation in clinically relevant models.

## 4 Structural modifications and derivatives of oleanolic acid

### 4.1 Semi-synthetic derivatives with enhanced cytotoxicity

To address the poor water solubility and low bioavailability of OA, chemical structural modifications are commonly employed to improve its physicochemical properties, enhance pharmacological activity, and optimize clinical applicability (Triaa et al., 2024; Verma et al., 2024; Tan et al., 2022). Structural modification represents a key strategy for improving the druggability of OA (Wang W. et al., 2022; Yang et al., 2024), as targeted alterations of the parent scaffold can yield derivatives with significantly enhanced bioactivity. Semi-synthetic OA derivatives such as SZC014, SZC015, HIMOXOL, and Br-HIMOLID have demonstrated superior anticancer activity compared with the parent compound (Chu et al., 2017; Lisiak et al., 2014; Lisiak et al., 2021; Wu et al., 2016). SZC014 inhibits proliferation, migration, and colony formation in MCF-7 breast cancer cells in a dose-dependent manner, inducing G_1_-phase cell cycle arrest and mitochondria-dependent apoptosis. Its pro-apoptotic effect is mediated by ROS generation and can be reversed by ROS scavengers (Chu et al., 2017). SZC015 exhibits even stronger antiproliferative effects in MCF-7 cells, simultaneously inducing apoptosis and autophagy. This activity involves multiple signaling pathways, including PI3K/Akt/mTOR, NF-κB, and MAPK, and is accompanied by inhibition of topoisomerase I/IIα activity (Wu et al., 2016). HIMOXOL exerts significant cytotoxicity in TNBC cells by activating both extrinsic apoptosis and autophagy pathways (Lisiak et al., 2014). Meanwhile, Br-HIMOLID targets HER2-positive breast cancer cells by binding HER2, activating the mTOR/LC3/SQSTM1/BECN1 autophagy pathway, and inhibiting Integrin β1/FAK/paxillin-mediated migration (Lisiak et al., 2021).

### 4.2 Glycosylated and saponin derivatives

Glycosylation represents another important strategy for optimizing the bioactivity of OA (Konishi et al., 2017; Vinh et al., 2020). By introducing sugar moieties at specific sites, glycosylation can significantly influence both cytotoxicity and mechanism of action (Staudacher et al., 2022). AH-Me exhibits notable cytotoxicity in MCF-7 and MDA-MB-453 cells, exerting its effects through carbomethoxy groups on the sugar moiety, which promote caspase-dependent apoptosis (Fukumura et al., 2009). Moreover, novel methoxylamino glycoside derivatives generated via glycosylation at the C-3 and C-28 positions demonstrate excellent antiproliferative activity. Among these, compound 8a shows the greatest sensitivity in HepG2 cells, inducing G0/G1-phase cell cycle arrest and apoptosis. These findings highlight that the type of sugar, its D/L configuration, and the site of attachment are critical determinants of biological activity (Du et al., 2021).

### 4.3 Electrophilic warheads and heterocyclic fusion

Incorporating electrophilic moieties or heterocyclic units into the OA scaffold is an effective strategy for developing novel derivatives with enhanced bioactivity (Bisseret et al., 2018; Péczka et al., 2022). Derivatives such as Y03/Y04, which introduce electrophilic α,β-unsaturated ketone groups into the A-ring of OA, can induce ROS generation and ferroptosis, while simultaneously inhibiting the Akt/mTOR signaling pathway and downregulating Bcl-2/Bcl-xL expression (Wang et al., 2023). A-ring fused heterocyclic derivatives, such as the 2-aminopyrimidine derivative 10, exhibit remarkably high antiproliferative activity in MCF-7 cells and are capable of inducing S-phase cell cycle arrest and apoptosis (Mallavadhani et al., 2014). Additionally, hydrazine and hydrazone derivatives at the C-3/C-28 positions display distinct activities: hydrazine derivatives retain antitumor potency, whereas hydrazone derivatives show diminished activity (Oyedeji et al., 2014).

### 4.4 Structure-activity relationship (SAR) insights

Systematic SAR studies have elucidated the intrinsic correlations between the structural features of OA derivatives and their anti-breast cancer activities, providing critical guidance for rational drug design (Polishchuk, 2017; Saganuwan, 2024). SAR analyses indicate that C-3 sulfonyl carbamate derivatives can significantly inhibit breast cancer cell migration and invasion at low micromolar concentrations, with their mechanism involving suppression of the Brk/Paxillin/Rac1 signaling pathway (Elsayed et al., 2015). Molecular docking studies reveal that Br-HIMOLID binds strongly to the HER2 kinase domain, explaining its high selectivity in HER2-positive cells (Lisiak et al., 2021). The activity of glycosylated derivatives is influenced by the sugar type and D/L configuration, and rational design of the sugar moiety can markedly enhance anticancer potency (Du et al., 2021).

## 5 Nanodelivery systems for oleanolic acid in breast cancer

### 5.1 Carrier-free nanomedicine

In recent years, carrier-free nanomedicines have attracted considerable attention due to their high drug-loading efficiency and excellent biocompatibility (Fu et al., 2022; Zheng et al., 2022; Zhong et al., 2023). In parallel, lipid-based co-delivery systems also provide an important framework for enhancing intracellular delivery and mechanistic synergy. For example, a decorated nanostructured lipid carrier designed for the simultaneous active targeting and co-delivery of three anticancer agents showed enhanced cellular internalization in folate receptor positive MCF-7 cells, accompanied by synergistic cytotoxicity and the involvement of ROS and autophagy related responses (Mahoutforoush et al., 2021). Building on these advances, OA can self-assemble with the photosensitizer Ce6 to form OC nanosensitizers, enabling synergistic chemotherapy and sonodynamic photodynamic therapy (Zheng et al., 2021). The resulting OC nanoparticles are approximately 100 nm in diameter, exhibit high photostability, and demonstrate improved tumor penetration, thereby enhancing intracellular Ce6 accumulation in cancer cells. *In vitro*, OC under combined laser and ultrasound irradiation induces ROS generation, mitochondrial membrane potential loss, apoptosis, and cell cycle arrest. In a 4T1 breast cancer mouse model, the combined treatment achieved a tumor inhibition rate of 82.5% with minimal systemic toxicity, highlighting the potential of carrier-free nanomedicines for synergistic cancer therapy.

### 5.2 Co-delivery with chemotherapeutics

Advanced formulation strategies for the co-delivery of OA with chemotherapeutic agents can markedly improve the *in vivo* pharmacokinetics, enhance tumor targeting, and generate synergistic antitumor effects (He et al., 2016; Liang et al., 2021). For instance, Put-D + O@NCs, a nanocrystal capsule anchored with putrescine, enables the co-delivery of Dox and OA, increasing intracellular drug accumulation in MDA-MB-231 cells, inducing G_2_/M phase arrest and apoptosis, while simultaneously inhibiting angiogenesis and MMP activity, thereby suppressing tumor metastasis (Shukla et al., 2021). Pharmacokinetic studies indicate that this system prolongs circulation time and enhances tumor accumulation, resulting in significant tumor inhibition in 4T1 xenografts with low toxicity. Similarly, chitosan-modified PLGA nanoparticles (CH-OA-B-PLGA) co-deliver OA and natural bioenhancers, improving cellular uptake, overcoming P-glycoprotein (P-gp) - mediated drug resistance, and inducing ROS-dependent apoptosis (Sharma et al., 2017). In 4T1 breast cancer mouse models, this system exhibits potent tumor suppression while protecting reproductive function, achieving a balance between therapeutic efficacy and fertility preservation.

## 6 Overcoming multidrug resistance and tumor microenvironment

### 6.1 Reversal of drug resistance

One of the central strategies for overcoming multidrug resistance is the inhibition of drug efflux pumps and the activation of noncanonical cell death pathways (Vasan et al., 2019). OA has been shown to significantly reduce ABCB1 (P-gp) expression in resistant tumor cells, thereby reversing drug resistance in MCF-7/Dox breast cancer and HL-60/HAR leukemia cells (Wang et al., 2021). OA induces G_1_ phase arrest in resistant cells and triggers non-classical apoptosis or alternative cell death mechanisms, demonstrating its potential to overcome MDR. A chitosan-modified PLGA co-delivery system (CH-OA-B-PLGA) further enhances OA accumulation in resistant cells by inhibiting P-gp efflux, achieving multi-modal apoptosis induction. In breast cancer mouse models, this system exhibits potent tumor suppression with minimal systemic toxicity and preserves reproductive function (Sharma et al., 2017).

### 6.2 Tumor microenvironment modulation

Targeting the tumor microenvironment, particularly by inhibiting angiogenesis and disrupting cancer stem cell niches, is an effective approach to suppress tumor progression and metastasis (Bader et al., 2020; Hinshaw and Shevde, 2019; Nagarsheth et al., 2017). Beyond passive accumulation, receptor-mediated targeting can further enhance tumor-specific internalization and improve the precision of microenvironment-responsive delivery. In this regard, dermatan sulfate-recognized UiO-66 metal-organic frameworks have been reported to achieve CD44-dependent uptake in MCF-7 cells, providing mechanistic evidence that MOF-based platforms can leverage receptor recognition to promote tumor-selective internalization and thereby strengthen the rationale for targeted delivery strategies (Mahoutforoush et al., 2025). Building on this concept, Put-D + O@NCs, a putrescine-anchored nanocrystal capsule co-delivering Dox and OA, not only induces apoptosis and G_2_/M phase arrest in cancer cells but also significantly inhibits angiogenesis, reduces MMP-2/9 expression and activity, and thereby suppresses tumor metastasis (Shukla et al., 2021). Furthermore, the small GTPase Rab13 is highly expressed in breast cancer stem cells (BCSCs), where it regulates CXCR1/2 receptor membrane trafficking, promotes tumor–stroma signaling, maintains stemness, and enhances chemoresistance (Sahgal et al., 2019; Wang H. et al., 2022). Targeting Rab13 may disrupt the BCSC niche and improve chemotherapy sensitivity, offering a promising direction for tumor microenvironment modulation.

Beyond angiogenesis and cancer stem cell niches, the immune compartment of the breast tumor microenvironment is a key determinant of response to immune checkpoint inhibitors (ICIs) targeting the PD-1/PD-L1 axis, and immunosuppressive myeloid programs such as tumor-associated macrophages can limit durable benefit (Debien et al., 2023; Jin et al., 2024; Cortes et al., 2022). Although direct evidence for OA–ICI combinations in breast cancer remains limited, emerging studies suggest that OA can modulate immune-relevant pathways, including cytokine signaling in immunocompetent breast tumor models and regulation of PD-L1 expression in cancer cells, providing a rationale to evaluate OA-based agents as immunomodulatory adjuvants rather than stand-alone immunotherapies (He et al., 2024; Lu et al., 2021). Future work should therefore test OA or representative derivatives in combination with ICIs in immunocompetent breast cancer models and quantify TME readouts such as CD8^+^ T-cell function, myeloid polarization, and PD-L1 dynamics alongside exposure and safety metrics (Li et al., 2021; Pu and Ji, 2022).

## 7 Chemoprevention potential of oleanolic acid

OA demonstrates differential redox-regulatory effects in normal versus cancerous cells, highlighting its potential as a chemopreventive agent (Nelson et al., 2015). In non-cancerous human mammary epithelial cells MCF10A, OA exhibits pronounced antioxidant properties, reducing intracellular ROS levels and protecting cells from H_2_O_2_-induced DNA damage (Sánchez-Quesada et al., 2015). In contrast, in highly invasive MDA-MB-231 breast cancer cells, OA exerts a pro-oxidant effect, elevating ROS production, inhibiting cell proliferation, and inducing DNA damage, thereby mediating its antitumor activity through oxidative stress mechanisms.

The oncogene KRAS is a critical driver of malignant transformation, conferring uncontrolled proliferation, survival, and metastatic potential (Molina-Arcas and Downward, 2024; Timar and Kashofer, 2020). Studies show that OA can non-cytotoxically induce autophagy in various normal human cells, evidenced by increased LC3-II levels and GFP-LC3 puncta formation. In KRAS-mutant transformed cells, OA-induced autophagy significantly suppresses proliferation, invasion, and anchorage-independent growth, while the autophagy inhibitor 3-methyladenine can partially reverse these effects. *In vivo*, OA delays tumor formation by KRAS-transformed MCF10A cells in mice, an effect dependent on autophagy induction. Mechanistically, OA triggers protective autophagy via inhibition of the Akt/mTOR/S6K signaling pathway, whereas IGF-1-mediated reactivation of this pathway blocks OA-induced autophagy (Liu et al., 2014a). In summary, OA exhibits a “bidirectional regulatory” property: it protects DNA in normal cells through antioxidant mechanisms, while in precancerous or cancer cells, it suppresses malignant transformation and tumor growth via pro-oxidant and autophagy-mediated mechanisms. These features underscore its potential as a natural chemopreventive and adjuvant therapeutic agent for breast cancer.

## 8 Discussion

Among natural compounds, pentacyclic triterpenoids are frequently explored as bioactive scaffolds for anticancer drug discovery (Li et al., 2023; Salvador et al., 2012). Within this class, OA has been investigated in breast cancer mainly in preclinical settings, where it has been associated with multi-layered regulation of cancer-relevant processes, including apoptosis, autophagy, ferroptosis, cell cycle control, redox homeostasis, and metabolic reprogramming (Baer-Dubowska et al., 2021; Zeng et al., 2024). Mechanistically, modulation of ROS levels, activation of AMPK, and inhibition of the PI3K/Akt/mTOR axis have been repeatedly implicated, supporting a pleiotropic mode of action that may influence tumor growth and treatment response (Table 1). However, it is important to emphasize that much of this mechanistic evidence is derived from a limited set of cell lines and experimental conditions, and causal validation is often incomplete, which constrains the generalizability of these conclusions across clinically diverse breast cancer subtypes.

**TABLE 1:** Overview of oleanolic acid and its derivatives: molecular targets, major effects, and study models.

Oleanolic acid or its derivative	Molecular targets	Major effects	Study models	Ref.
SZC014	ROS ↑, Akt ↓, p-Akt ↓, COX-2 ↓, p-p65 (cytoplasm) ↓, p65 (nucleus) ↓, Nuclear translocation of p65 (inhibited), Bax/Bcl-2 ratio ↑, Procaspase-9 ↓, Procaspase-3 ↓, PARP cleavage ↑	Inhibits cell proliferation and viability, Induces apoptosis, Suppresses cell migration and colony formation, Induces G_1_ phase cell cycle arrest, Effects are ROS-dependent	*In vitro*: Human breast cancer cell lines MCF-7, MDA-MB-231; human mammary epithelial cell line MCF10A	Chu et al. (2017)
Achyranthoside H methyl ester (AH-Me)	PARP cleavage ↑, Caspase-9 ↑	Induces apoptosis, Inhibits cell proliferation, Cytotoxic to breast cancer cells	*In vitro*: Human breast cancer cell line MCF-7, MDA-MB-453	Fukumura et al. (2009)
Oleanolic Acid	Bax/Bcl-2 ratio ↑, Caspase-8 ↑, ROS ↑, Mitochondrial Membrane Potential ↓, Cyclin D1 ↓, Cyclin E1 ↓	Induces G1 cell cycle arrest, Induces apoptosis (intrinsic and extrinsic pathways), Inhibits cell proliferation, Generates ROS	*In vitro*: Human breast cancer cell line MCF-7, MDA-MB-231	Chakravarti et al. (2012)
BN107	mTOR/FRAP1 ↓, RAPTOR ↓, RICTOR ↓, Akt (phospho-Ser473) ↓	Induces apoptosis selectively in ER- breast cancer cells by concomitant inhibition of mTORC1 and mTORC2 activities through cholesterol depletion in lipid rafts	*In vitro*: Human breast cancer cell line MDA-MB-231, HS578T, MCF7	Chu et al. (2010)
HIMOXOL	Bax/Bcl-2 ratio ↑, Caspase-8 ↑, Caspase-3 ↑, Cleaved PARP-1 ↑, LC3-II ↑, Beclin 1 ↑, p-p38α ↑, p38α ↑, JNK1/2 ↓, p-JNK1 ↓, Nuclear p53 ↑, NF-κB (p65) ↓	Induces apoptosis (extrinsic pathway), Induces autophagy, Reduces cell viability, Triggers dual cell death (apoptosis and autophagy)	*In vitro*: Human breast cancer cell line MDA-MB-231	Lisiak et al. (2014)
Oleanolic acid	p-Akt ↓, p-mTOR ↓, p-S6K ↓	Induced autophagy, inhibited proliferation, invasion, and anchorage-independent growth of KRAS-transformed cells	*In vitro*: Human breast cancer cell line MCF10A *In vivo*: MCF10A xenograft in mice	Liu et al. (2014a)
HIMOXOL, Br-HIMOLID	mTOR ↓, LC3-II/LC3-I ratio ↑, BECN1 ↑, SQSTM/p62 ↓, ITGB1 ↓, p-FAK (Tyr397) ↓, PXN ↓	Induced autophagy, decreased cell viability, cytostatic effect (G0/G1 arrest), anti-migratory activity	*In vitro*: Human breast cancer cell line SK-BR-3	Lisiak et al. (2021)
ZQL-3d, 27a, Y03, Y04	p-Akt ↓, p-mTOR ↓, Bcl-2 ↓, Bcl-xL ↓, Cyclin D1 ↓, Cyclin B1 ↓, GPX4 ↓	Cytotoxic activity, inhibits proliferation and migration, induces apoptosis and G0/G1 arrest, promotes ROS generation, induces ferroptosis	*In vitro*: Human breast cancer cell line MCF-7, MDA-MB-231	Wang et al. (2023)
SZC015	Bax ↑, Bcl-2 ↓, Cyt C (cytosolic) ↑, caspase-3 ↓, caspase-9 ↓, PARP ↓, c-PARP ↑, LC3-II/LC3-I ↑, Atg5 ↑, Beclin1 (10/20 μM ↑, 30 μM ↓), PI3K (p110, p85) ↓, p-Akt/Akt ↑, p-mTOR ↓, p-IκBα/IκBα ↓, NF-κB p65 (CF/NF) ↓, p-p38α/p38α ↓, p-JNK1/JNK1 ↓, p-ERK1/2/ERK1/2 ↓, Top I ↓, Top IIα ↓	Induces apoptosis and autophagy, causes S-phase cell cycle arrest, inhibits PI3K/Akt/mTOR/NF-κB and MAPK pathways, inhibits topoisomerase activity	*In vitro*: Human breast cancer cell line MCF-7	Wu et al. (2016)
Oleanolic acid	ROS ↑, CAT ↑, CAT ↓	Inhibits proliferation of highly invasive breast cancer cells, reduces oxidative stress and DNA damage in normal mammary epithelial cells	*In vitro*: Human mammary epithelial cell line MCF10A, human breast cancer cell line MDA-MB-231, MCF7	Sánchez-Quesada et al. (2015)
Oleanolic acid	p-mTOR (Ser2448) ↓, mTOR ↓, c-Myc ↓, hnRNPA1 ↓, hnRNPA2 ↓, PKM2 ↓, PKM1 ↑	Suppresses aerobic glycolysis, induces PKM2 to PKM1 isoform switch, inhibits cancer cell proliferation and colony formation	*In vitro*: Human breast cancer cell line MCF7	Liu et al. (2014b)
Oleanolic acid	p-AMPKα (Thr172) ↑, p-ACC (Ser79) ↑, FASN ↓, p-HMGR (Ser872) ↑, p-mTOR (Ser2448) ↓, p-p70S6K ↓, p-4E-BP1 ↓	Activates AMPK pathway, suppresses lipogenesis, protein synthesis and aerobic glycolysis, inhibits cancer cell proliferation and colony formation, induces cell cycle arres	*In vitro*: Human breast cancer cell line MCF7	Liu et al. (2014c)
Oleanolic acid (1), 3-O-[N-(3'-chloro-benzenesulfonyl)-carbamoyl]-oleanolic acid (11), 3-O-[N-(5'-fluorobenzenesulfonyl)-carbamoyl]-oleanolic acid (12)	p-Brk ↓, p-Paxillin ↓, p-Rac1 ↓, p-Akt ↓, p-ERK1/2 ↓	Inhibits migration, proliferation, and invasion of highly invasive breast cancer cells, suppresses Brk/Paxillin/Rac1 signaling axis	*In vitro*: Human mammary epithelial cell line MCF10A, human breast cancer cell line MDA-MB-231	Elsayed et al. (2015)
CDDO-Me	Rab13↓	Inhibits breast cancer stem cell (BCSC) stemness, reduces tumor growth and chemoresistance, disrupts tumor-stroma cross-talk by decreasing TAM and CAF recruitment	*In vitro*: Human breast cancer cell line MDA-MB-231 *In vivo*: MDA-MB-231 xenograft in nude mice	Wang et al. (2022b)
Oleanolic acid, 3-acetoxyoleanolic hydrazide, 3-acetoxyoleanolic hydrazone	NA	OA and 3-acetoxyoleanolic hydrazide show cytotoxic activity against MCF-7 cells, while 3-acetoxyoleanolic hydrazone is inactive	*In vitro*: Human breast cancer cell line MCF7	Oyedeji et al. (2014)
28-N-methoxyaminoolenane-β-D-glucoside (8a) and other C-3/C-28 MeON-neoglycosides	NA	Cell cycle arrest at G0/G1 phase, Apoptosis induction	*In vitro*: Human breast cancer cell line MCF7	Du et al. (2021)

A major barrier to translation is OA’s suboptimal druggability (Wasim and Bergonzi, 2024). OA exhibits poor aqueous solubility and limited oral bioavailability, and its extensive metabolism may further reduce effective systemic exposure, making it difficult to establish consistent exposure–response relationships *in vivo* (Zhang et al., 2014; García-González et al., 2023; Chen et al., 2012). These pharmacokinetic liabilities are not merely formulation issues but fundamental constraints that can confound efficacy interpretation when *in vitro* active concentrations are not achievable in tumors. While semi-synthetic derivatives have been reported to improve potency and, in some cases, physicochemical properties (Chu et al., 2017; Lisiak et al., 2014; Lisiak et al., 2021), claims of improved pharmacokinetics and selectivity remain unevenly supported because comparative PK, biodistribution, and PK/PD linkage data are still scarce. Moreover, derivatives may introduce new risks, including altered metabolic fate, potential drug–drug interactions, and a narrower safety window, which are rarely evaluated systematically in long-term studies (D'Mello et al., 2025).

Nanodelivery systems and carrier-free self-assembly strategies are frequently proposed to overcome OA’s solubility and delivery limitations and to enhance tumor accumulation (Gao L. et al., 2024; Li et al., 2022). Although multiple platforms have shown antitumor activity with apparently low acute toxicity *in vitro* and in short-term *in vivo* studies (Shukla et al., 2021; Sharma et al., 2017), key translational hurdles remain. These include batch-to-batch reproducibility, scalable manufacturing, stability in biological fluids, immune interactions, off-target organ accumulation, and the lack of standardized reporting of critical quality attributes. Importantly, improved “tumor targeting” is often inferred from enhanced efficacy rather than demonstrated through quantitative biodistribution and tumor exposure measurements. Therefore, nanomedicine-based improvements should be interpreted cautiously until supported by rigorous PK, biodistribution, and long-term safety datasets.

The review also highlights promising but still preliminary directions such as OA-associated modulation of multidrug resistance and the tumor microenvironment, including angiogenesis and cancer stem cell niches (Shukla et al., 2021; Wang H. et al., 2022). However, the current literature is skewed toward positive findings, with limited reporting of negative or contradictory results, and insufficient validation in clinically relevant models such as patient-derived organoids, xenografts, and immunocompetent systems (Gao W. et al., 2024; Ritskes-Hoitinga and Pound, 2022; Shi et al., 2024). These limitations likely contribute to optimistic interpretations and underscore the need for more stringent and standardized preclinical evaluation.

To address the specific gaps identified throughout this review, future work should prioritize the following actionable directions. First, strengthen evidence quality by expanding testing across molecular subtypes and using appropriate normal breast epithelial controls, while adopting standardized dosing, time points, and endpoints to improve comparability (Souto et al., 2022; Villarreal-García et al., 2022). Second, establish robust pharmacokinetic foundations by performing head-to-head PK and biodistribution studies for OA, representative derivatives, and leading formulations, and by integrating PK/PD analyses to define exposure thresholds linked to mechanistic readouts and tumor response (Papachristos et al., 2023; Ruiz-Garcia et al., 2023). Third, implement systematic safety evaluation, including long-term toxicity, immunotoxicity, and metabolite profiling for derivatives, as well as comprehensive biocompatibility and clearance studies for nanocarriers (Csóka et al., 2021; Đorđević et al., 2022; Chen et al., 2023; Xu Zhao et al., 2024). Fourth, validate mechanistic causality using genetic or pharmacological perturbation strategies rather than correlative marker changes, and identify biomarkers that can bridge preclinical mechanisms to clinically measurable endpoints (Zhou Y. et al., 2024). Fifth, develop rational combination strategies guided by mechanistic hypotheses, such as pairing OA-based agents with standard chemotherapy, targeted therapy, or immunotherapy, and evaluate these combinations in immunocompetent models with tumor microenvironment and immune readouts (Emens and Loi, 2023; Harris et al., 2024). Finally, for nanomedicines, define clear translational criteria including manufacturability, reproducibility, stability, and regulatory-relevant quality attributes, so that efficacy claims are aligned with practical feasibility (Taha et al., 2020).

In conclusion, OA and its derivatives should be regarded as preclinical leads that illuminate potentially druggable mechanisms and delivery opportunities in breast cancer, rather than as established therapeutic or chemopreventive agents. A balanced interpretation of the field supports continued investigation, but meaningful clinical translation will require rigorous PK/PD characterization, standardized model validation, and comprehensive safety assessment.
